# Comparative analysis of cognitive impairment prevalence and its etiological subtypes in a rural area of northern China between 2010 and 2015

**DOI:** 10.1038/s41598-018-37286-z

**Published:** 2019-01-29

**Authors:** Hui Lu, Xiao-Dan Wang, Zhihong Shi, Wei Yue, Ying Zhang, Shuai Liu, Shuling Liu, Lei Zhao, Lei Xiang, Yajing Zhang, Yalin Guan, Wenhua Su, Zhiyong Li, Jinhuan Wang, Thomas Wisniewski, Yong Ji

**Affiliations:** 1 Department of Neurology, Tianjin Huanhu Hospital, Tianjin, China; 2 Tianjin Key Laboratory of Cerebrovascular and Neurodegenerative Diseases, Tianjin Huanhu Hospital, Tianjin, China; 3 Tianjin Jixian County Health Bureau, Tianjin, China; 4 New York University School of Medicine, Department of Neurology, Psychiatry and Pathology, New York, N.Y. USA

## Abstract

The purpose of this study was to evaluate changes in the prevalence and risk factors of cognitive impairment (CI) by analyzing and comparing two cross-sectional epidemiological surveys of CI and its subtypes were performed in a rural area of northern China between 2010 and 2015. Residents aged ≥60 years were drawn in northern China. The Mini Mental State Examination (MMSE) is recommended to test for CI. Dementia was further categorised into Alzheimer’s disease (AD), vascular dementia (VaD), and dementia caused by other diseases (ODs). Mild cognitive impairment (MCI) was classified into MCI caused by AD (MCI-A), MCI caused by VaD (MCI-VD), and MCI caused by ODs (MCI-O). The prevalence of CI increased in China. The prevalence of all-cause CI was 30·5% (22.9% MCI and 7.6% dementia) in 2010. The prevalence of all-cause CI was 38.3% (27.8% MCI and 10.5% dementia) in 2015. Similar increases were observed for the prevalence of subtypes of dementia and MCI. These findings suggest an increasing prevalence of CI and its subtypes in China, which may be related to alterations in sociodemographic factors, vascular risk factors and lifestyle changes over time in these cohorts.

## Introduction

Population aging changes lifestyle habits and public health problems. Chronic non-communicable diseases have become the leading cause of mortality in China. Dementia is one of these non-communicable diseases that is considered to have the greatest economic and social effects, particularly in rural regions^1,2^. The World Alzheimer Report 2010^3^ predicted that dementia will have greater economic effect than that of cancer, heart disease, and stroke combined.

Assessing cognitive impairment (CI) is an important part of the diagnostic criteria for dementia. CI ranges from mild to severe, and is one of the most common and disabling non-motor symptoms among elderly individuals. The prevalence of severe CI (dementia) is reported to be approximately 4−8% in Western developed countries and 5−7% in China^4^. The number of people with dementia is likely to increase as the population ages^5^. Mild cognitive impairment (MCI), which is also designated as cognitive impairment no dementia (CIND), is used to describe syndromes in older adults that include a broad array of cognitive impairments. MCI represents an intermediate stage between normal aging and dementia. Patients with MCI have a much higher risk of dementia. After an initial diagnosis of MCI, the incidence of dementia within 1, 5, and 9.5 years was 10–15, 60.5 and 100%, respectively^6^. Early identification of subjects at risk for MCI is a key interventional target for dementia, and adopting effective preventive strategies may delay or even prevent dementia. Therefore, MCI has received increased attention in recent decades.

There were some studies on the risk factors for CI in Chinese populations. Older age was the risk factors for dementia^7–9^. Female was the risk factor for AD, hypertension was the risk factor for VaD^9^. However, another study found there is no difference in risk between men and women^7^. Illiteracy was the risk factor of dementia in rural Chinese population^8,9^. Living without a partner, female and previous stroke were risk factors for CIND^10^. However, there is no study on the risk factors for the trend of prevalence of CI and CIND in rural Chinese populations.

Understanding the epidemiology of CI and its subtypes in a given population is crucial for adequate planning of public health strategies and rational allocation of resources. The identification of subjects at risk for CI is important for the implementation of potential treatments that may delay or prevent cognitive decline. However, population-based data monitoring for predicting the prevalence of CI and its subtypes remains scarce. As the population ages, we hypothesize that the prevalence rate of CI increases and its etiological subtypes might change from 2010 to 2015. Therefore, the present study was performed to survey the prevalence rate of CI and its etiological subtypes in Ji County, China, using uniform identification and methodology protocols and data from epidemiological surveys on CI in China conducted in 2010 and 2015. The comparative analysis aims to estimate trends in the prevalence of CI and its etiological subtypes during the intervening 5 years, and to describe the various factors associated with CI, which can change over time.

## Methods

### Subjects

The participants were chosen from the database of Health Bureau of Ji County. Every investigation randomly selected 62 villages belonging to the four townships of Ji County, which is 110 km away from Tianjin (located in northern China) and have similar environment, dietary and living habits, a low population mobility rural area in a mountainous region. Most of the population is composed of Han Chinese, who are farmers with low education levels and low incomes. The following inclusion criteria were used: subjects were aged ≥60 years, and were legal residents in Ji County for at least 5 years preceding the survey date who accepted to participate. Exclusion criteria were hearing loss, refusal to participate in the study, retirement of the consent to participate, death or migration (23 individuals in 2010 and 51 individuals in 2015 were unsurveyed) . The study included a total of 5,581 individuals in 2010 (2,484 men and 3,097 women, with mean age 69.83 ± 7.27 years) and 5,542 individuals in 2015 (2,564 men and 2,978 women, with mean age 70.50 ± 7.84 years). We did door-knocking investigate of all participants leading by country doctors of each village. The study was approved by the Committee for Medical Research Ethics at Tianjin Huanhu Hospital and Tianjin Health Bureau. Informed consent was directly obtained from each subject, or was indirectly obtained from the subject’s guardian. We confirm that all methods were performed in accordance with the relevant guidelines and regulations by including a statement in the methods section to this effect.

### Assessment and Diagnostic Procedures

All interviewers were junior neurologists or senior graduate students specializing in neurology, who received uniform training on neuropsychological assessment and diagnosis for 1 week, and participated in a retraining course every 3 months thereafter. A two-stage method was applied in the principle investigation. First, the interviewed subjects were contacted directly during a home visit. They were informed of the objective of the interview and welcomed to participate. Consent was obtained, and the home interview was conducted by a minimum of two members from a team of 10 local practitioners who had obtained their medical licenses more than 5 years before the study. The practitioners were selected because of their willingness to participate in the study and their ability to complete the home visits. The whole team was trained as a group by two neurologists who specialize in dementia and Alzheimer’s disease from the Tianjin Dementia Institute at Tianjin Huanhu Hospital. The training included how to collect information and how to evaluate cognitive impairment. Data was collected for each subject for the demographic questionnaire (age, gender and education), personal history (smoking more than half a year including past smokers and current smokers and drinking liquor everyday including past drinkers and current drinkers) and medical history (Cardiovascular disease or heart failure, diabetes, hypertension and obesity) and a brief physical and neurological examination. Age was divided into three stages: 60–69 years, 70–79 years and equal or greater than 80 years. Education included illiterate, primary school and middle school and above. Then, all subjects were asked to complete the Chinese Mini Mental State Examination. Reliable informants (including spouses, children, other relatives and close friends, in descending order) were asked to complete the Activities of Daily Living (ADL) and Clinical Dementia Rating questionnaires to help provide necessary information if the subjects could not provide the information themselves. Subjects with a Chinese Mini Mental State Examination score under the cutoff points (i.e., 17 for illiterate persons, 20 for persons with 1–6 years of education, and 24 for persons with ≥7 years of education) and/or a Clinical Dementia Rating judged to be ≥0.5 were deemed eligible for the second stage of the study. The average duration of an interview was 20 min. Finally, positive subjects were examined to confirm or exclude the presence of dementia or MCI. A home interview was performed again, but this time, two of six board-certified neurologists from the Dementia Center at Tianjin Huanhu Hospital performed the assessment. The six neurologists were trained together to ensure uniform neurological assessments across participants. A detailed medical history, physical examination and neurological examination were undertaken for each subject.

Subjects were classified into three general categories: normal, MCI and dementia. A diagnosis of dementia was made based on the Diagnostic and Statistical Manual of Mental Disorders (IV Edition) criteria: the participant must present the development of multiple cognitive deficits including memory impairment and impairment in at least one other cognitive domain, which represents a decline from the previous level of functioning with sufficient severity to cause impairment in function. MCI was diagnosed as: (1) cognitive complaint that did not meet the criteria for dementia, (2) scores on the Mini Mental State Examination (MMSE) below 27 and did not meet the scores of dementia, and (3) intact ADL.

Dementia was further categorized into Alzheimer’s dementia (AD), vascular dementia (VaD) and dementia resulting from other diseases (DM-O). MCI was further categorized into MCI resulting from Alzheimer’s disease (MCI-A), MCI resulting from vascular disease (MCI-VD) and MCI resulting from other diseases (MCI-O). The diagnosis of AD and VaD were made based on the Diagnostic and Statistical Manual of Mental Disorders (IV Edition) criteria. The VaD participant must conform to the dementia criteria and present focal neurological signs and symptoms or neuroimagen evidence. The other types of dementia could not attribute to AD or VaD were diagnosed as DM-O. Etiological diagnoses of MCI depend on the medical history and cognitive impairment characteristics. Significant memory impairment and having no or less vascular risk factors mean MCI-A. Significant excutive function impairment and having tight relationship with cerebral vascular disease mean MCI-VD. The other MCI was diagnosed as MCI-O.

### Statistical analyses

The raw prevalence of MCI and dementia and their subtypes was determined by calculating the total number of cases of MCI and dementia and their subtypes with respect to the total number of study participants. The 95% confidence intervals (95% CI) also were computed.

Continuous variables were expressed as the mean ± standard deviation (SD), whereas qualitative variables were expressed as frequency distributions. Logistic regression analysis was used to evaluate independent associations between the presence of MCI/dementia and sociodemographic factors, vascular risk factors, and lifestyle habits. The data was analyzed using the SPSS/PC + version 18.0 statistical package. Odds ratios (ORs) were calculated for each variable, and a significance level of *P*<0·05 was required to retain a variable in the model.

## Results

### Characteristics of the study population

Table 1 presents the characteristics of the study populations in 2010 and 2015. Compared with the 2010 cohort, the 2015 cohort was slightly older (mean of 70.50 years versus 69.83 years), had significantly more illiteracy and included fewer individuals with diplomas from middle school or higher education. Illiterate individuals comprised 39.0% of the 2015 cohort and 36.6% of the 2010 cohort. Individuals with middle school and higher education comprised 15.6% of the 2015 cohort and17·8% of the 2010 cohort. However, there was no significant difference in gender in 2010 and 2015. The 2015 cohort had fewer single subjects (no spouse), but significantly higher levels of social activities. There was a lower rate of obesity in the 2015 cohort, but higher rates of vascular risk factors including heart disease, diabetes, and hypertension. The 2015 cohort had higher levels of tobacco and alcohol consumption.

**Table 1:** Characteristics of the 2010 and 2015 study cohorts.

Total	2010	2015	Statistics	P*
	5581	5542		
**Age (years)**			49.281	0.000
60−69	3078 (55.2)	2882 (52.0)		
70−79	1858 (33.3)	1765 (31.8)		
≥80	645 (11.6)	895 (16.1)		
**Mean ± SE**	69.83 ± 7.27	70.50 ± 7.84	−4.668	0.000
**Gender**			3.462	0.063
Male	2484 (44.5)	2564 (46.3)		
Female	3097 (55.5)	2978 (53.7)		
**Education**			12.951	0.002
Illiterate	2040 (36.6)	2160 (39.0)		
Primary school	2546 (45.6)	2520 (45.5)		
Middle school and above	995 (17.8)	862 (15.6)		
Widowed/Separated/Single	1005 (18.0)	851 (15.4)	14.069	0.000
No/little social activity	2457 (44.0)	958 (17.3)	934.398	0.000
**Vascular risk factors**				
Heart disease	509 (9.1)	638 (11.5)	17.201	0.000
Diabetes	392 (7.0)	530 (9.6)	23.590	0.000
Hypertension	2100 (37.6)	2242 (40.5)	9.339	0.002
Obesity	548 (9.8)	119 (2.1)	290.337	0.000
**Lifestyle habit**				
Smoking	1381 (24.7)	1633 (29.5)	31.376	0.000
Alcohol consumption	971 (17.4)	1280 (23.1)	55.931	0.000

*P value for chi-square or *t*-test for significant difference in proportion or mean between years.

### Trends in the prevalence of CI and CI subtypes and adjusted odds ratios of AD, VaD, and MCI-VD

We calculated the prevalence of CI and CI subtypes for 2015 and 2010. In 2010, the prevalence of all-cause CI was 30·5% (22.9% MCI and 7.6% dementia), and in 2015 the prevalence of all-cause CI was 38.3% (27.8% MCI and 10.5% dementia). Therefore, we found that the prevalence of CI in the 2015 cohort was significantly higher than that of the 2010 cohort. Similar increases were observed for 2010 and 2015 data on AD (5.1 and 6.5%, respectively), VaD (1.7 and 2.3%, respectively), ODs (0.9 and 1.7%, respectively), MCI-VD (2.3 and 6.8%, respectively), and MCI-O (1.6 and 2.7%, respectively). However, there was no significant difference for MCI-A data in 2010 and 2015 (19.0 and 18.4%, respectively).

We used pooled 2010 and 2015 data to run six different logistic regression models with trend in AD prevalence as the outcome variable (Table 2). Table 2 shows the odds ratio in AD trends from 2010 to 2015 in the first row, and then adjusts for age and gender (Model 2), education level (Model 3), no spouse and no/infrequent social activity (Model 4), vascular risk factors (Model 5), and lifestyle habits (Model 6). Our analysis showed that older age, female gender, single status (no spouse), no/infrequent social activity, and hypertension were the risk factors that accounted for the increased prevalence of AD in 2015. The prevalence also increased with age. On the other hand, higher education level and alcohol consumption were protective factors for AD, and the trend decreased with higher education. The presence of heart disease, diabetes, obesity, and smoking had no significant influence on the increasing AD trend.

**Table 2:** Odds ratios for the trend in prevalence of Alzheimer’s dementia from 2010 to 2015(N = 11,123).

Variable	Model 1	Model 2	Model 3	Model 4	Model 5	Model 6
Trend (2015/2010)	1.451 (1.234–1.707)	1.278 (1.065–1.533)	1.370 (1.138–1.650)	1.874 (1.531–2.293)	1.769 (1.440–2.172)	1.804 (1.468–2.217)
**Age**						
60–69		Ref.	Ref.	Ref.	Ref.	Ref.
70–79		3.540 (2.811–4.457)	3.070 (2.431–3.877)	2.721 (2.146–3.450)	2.664 (2.100–3.381)	2.686 (2.116–3.409)
80+		32.510 (25.667–41.176)	21.563 (16.865–27.568)	15.373 (11.839–19.962)	14.963 (11.491–19.484)	14.894 (11.432–19.403)
Female gender		2.705 (2.232–3.278)	1.934 (1.579–2.368)	1.832 (1.492–2.249)	1.750 (1.421–2.156)	1.498 (1.181–1.900)
**Education**						
Illiterate			Ref.	Ref.	Ref.	Ref.
Primary school			0.416 (0.339–0.511)	0.439 (0.356–0.540)	0.425 (0.344–0.525)	0.430 (0.348–0.531)
Middle school and above			0.183 (0.121–0.275)	0.199 (0.132–0.300)	0.196 (0.130–0.296)	0.196 (0.130–0.296)
Widowed/Separated/Single				1.485 (1.191–1.852)	1.461 (1.170–1.823)	1.447 (1.159–1.806)
No/Little social activity				2.437 (1.991–2.983)	2.439 (1.990–2.989)	2.448 (1.997–3.001)
**Vascular Risks**						
Heart disease					0.875 (0.639–1.196)	0.878 (0.641–1.201)
Diabetes					0.819 (0.559–1.200)	0.806 (0.550–1.181)
Hypertension					1.889 (1.561–2.287)	1.891 (1.562–2.290)
Obesity					0.762 (0.465–1.247)	0.763 (0.466–1.249)
**Living habit**						
Smoking						0.971 (0.737–1.279)
Alcohol consumption						0.643 (0.464–0.892)

We also used pooled 2010 and 2015 data to run six different logistic regression models with trends in VaD (Table 3) and MCI-VD (Table 4) as output variables. The results showed that older age, no/infrequentsocial activity, diabetes, hypertension and obesity were risk factors for VaD and MCI-VD; higher education level and alcohol consumption were protective factors; and smoking did not significantly affect VaD and MCI-VD. Heart disease and single status (no spouse) did not significantly affect VaD, and female gender was a protective factor for VaD. For MCI-VD, female gender, single status (no spouse) and heart disease were risk factors.

**Table 3:** Odds ratios for the trend in prevalence of VaD from 2010 to 2015(N = 11, 123).

Variable	Model 1	Model 2	Model 3	Model 4	Model 5	Model 6
Trend (2015/2010)	1.507 (1.149–1.978)	1.405 (1.067–1.850)	1.494 (1.132–1.971)	2.140 (1.586–2.887)	2.352 (1.726–3.207)	2.433 (1.782–3.321)
Age						
60–69		Ref.	Ref.	Ref.	Ref.	Ref.
70–79		2.146 (1.572–2.930)	1.806 (1.317–2.477)	1.655 (1.201–2.282)	1.664 (1.202–2.305)	1.666 (1.203–2.307)
80+		7.316 (5.103–10.490)	4.891 (3.365–7.109)	3.643 (2.444–5.431)	4.237 (2.810–6.390)	4.255 (2.821–6.419)
Female gender		0.754 (0.570–0.998)	0.545 (0.406–0.731)	0.519 (0.386–0.699)	0.405 (0.297–0.553)	0.346 (0.245–0.487)
Education						
Illiterate			Ref.	Ref.	Ref.	Ref.
Primary school			0.386 (0.285–0.523)	0.405 (0.298–0.551)	0.367 (0.268–0.502)	0.371 (0.271–0.506)
Middle school and above			0.193 (0.114–0.324)	0.205 (0.121–0.346)	0.181 (0.106–0.307)	0.179 (0.105–0.305)
Widowed/Separated/Single				1.074 (0.738–1.561)	1.096 (0.751–1.599)	1.077 (0.738–1.573)
No/Little social activity				3.185 (2.361–4.296)	3.289 (2.429–4.452)	3.357 (2.477–4.549)
Vascular Risks						
Heart disease					0.920 (0.566–1.496)	0.924 (0.567–1.503)
Diabetes					3.232 (2.191–4.767)	3.204 (2.170–4.730)
Hypertension					1.865 (1.402–2.482)	1.868 (1.403–2.487)
Obesity					2.635 (1.584–4.382)	2.713 (1.629–4.520)
Living habit						
Smoking						1.032 (0.719–1.481)
Alcohol consumption						0.610 (0.408–0.910)

**Table 4:** Odds ratios for the trend in prevalence of MCI-VD from 2010 to 2015(*N* = 11, 123).

Variable	Model 1	Model 2	Model 3	Model 4	Model 5	Model 6
Trend (2015/2010)	3.330 (2.711–4.091)	3.304 (2.682–4.068)	3.541 (2.865–4.376)	3.904 (3.122–4.883)	3.780 (3.008–4.749)	3.836 (3.052–4.822)
Age						
60–69		Ref.	Ref.	Ref.	Ref.	Ref.
70–79		2.106 (1.720–2.578)	1.738 (1.448–2.195)	1.667 (1.349–2.059)	1.605 (1.296–1.988)	1.601 (1.292–1.984)
80+		5.136 (3.901–6.764)	3.113 (2.337–4.148)	2.627 (1.942–3.553)	2.623 (1.929–3.566)	2.653 (1.950–3.608)
Female gender		2.087 (1.719–2.533)	1.384 (1.129–1.698)	1.350 (1.100–1.657)	1.180 (0.956–1.457)	1.112 (0.869–1.422)
Education						
Illiterate			Ref.	Ref.	Ref.	Ref.
Primary school			0.293 (0.238–0.361)	0.301 (0.244–0.371)	0.280 (0.226–0.347)	0.282 (0.228–0.350)
Middle school and above			0.101 (0.065–0.158)	0.105 (0.067–0.165)	0.099 (0.063–0.155)	0.100 (0.063–0.157)
Widowed/Separated/Single				1.446 (1.129–1.852)	1.414 (1.101–1.816)	1.399 (1.089–1.797)
No/Little social activity				1.309 (1.041–1.648)	1.331 (1.056–1.677)	1.345 (1.067–1.696)
Vascular Risks						
Heart disease					1.383 (1.048–1.825)	1.386 (1.050–1.829)
Diabetes					1.451 (1.075–1.960)	1.437 (1.064–1.941)
Hypertension					2.270 (1.867–2.759)	2.273 (1.870–2.764)
Obesity					0.781 (0.460–1.326)	0.792 (0.466–1.345)
Living habit						
Smoking						0.792 (0.466–1.345)
Alcohol consumption						0.691 (0.503–0.948)

## Discussion

The first large population-based cross-sectional comparative surveys of CI in 2010 and 2015 identified a trend in increasing prevalence of CI and its etiological subtypes (AD, VaD, ODs, MCI-A, MCI-V and MCI-O) in rural areas of northern China. These surveys identified the risk factors associated with changes in the prevalence rates of MCI, dementia, and the etiological subtypes. The CI prevalence rate in 2015 was 38·3% (27.8% MCI and 10.5% dementia), which was significantly higher than the 30·5% prevalence in 2010 (22.9% MCI and 7.6% dementia). Further study of the CI etiological subtypes in 2010 and 2015 indicated similar increases in AD (5.1 and 6.5%, respectively), VaD (1.7 and 2.3%, respectively), ODs (0.9 and 1.7%, respectively), MCI-VD (2.3 and 6.8%, respectively), and MCI-O (1.6 and 2.7%, respectively). However, MCI-A did not significantly differ in 2010 and 2015 (19.0 and 18.4%, respectively). Chan *et al*.^11^ reported that the number of people with dementia in China was 3.68 million (95% CI 2.22–5.14) in 1990, 5.62 million (4.42–6.82) in 2000, and 9.19 million (5.92–12.48) in 2010. During the same period, the number of people with Alzheimer’s disease was 1.93 million (1.15–2.71) in 1990, 3.71 million (2.84–4.58) in 2000, and 5.69 million (3.85–7.53) in 2010. Jia J *et al*.^12^ reported that the prevalence of dementia, AD, and VaD among Chinese aged ≥65 years was 5.14, 3.21, and 1.50%, respectively. Ding D *et al*.^13^ reported that the prevalence of dementia, AD, and VaD among Chinese aged ≥60 years was 5.0, 3.6, and 0.8%, respectively. Reviews report CIND prevalence ranging from 5–29%^14,15^. Jia^16^ and Ding^17^ suggested that the prevalence of MCI was approximately 20%.

The present study revealed a remarkably higher prevalence of dementia and MCI compared to reports from other populations in China^12,13,16–19^, the United States (16.0–22.2%)^20^ and South Korea (24.1%)^21^, but similar prevalence to previous reports from some Western countries^22^. The higher prevalence of dementia and MCI in the present study may be explained by regional differences and different investigation methods. Moreover, low education of the population is possibly the cause of higher prevalence of dementia. The Subjects contacted directly by home visits tend to have higher prevalence of MCI than those examined by questionnaire survey. A large nationally representative survey of older Americans found a decline in CI prevalence, which suggested that the combined effects of recent trends in medical, lifestyle, demographic, and social factors had improved the cognitive health of older Americans^20^. The current population-based cross-sectional comparative survey across 5 years showed that the prevalence of AD, VaD, and MCI-VD were increasing, and there was a significant increase in the prevalence of CI caused by other factors such as Parkinson’s disease, frontotemporal lobar degeneration, Lewy body disease, and traumatic brain injury. The observed changes in the prevalence of most CI subtypes suggest that the combined impact of recent trends in medical, lifestyle, environmental, and social factors are affecting the cognitive health of older Chinese individuals.

Our analysis showed that older age was the risk factor accounting for the increasing prevalence of AD, VaD and MCI-VD, and the relevance of this risk factor increased with age. Age is the most important risk factor of AD and the prevalence increases with age^11^. This result is in agreement with previous studies showing that the rate of dementia approximately doubles every 5 years between the ages of 70 and 84^23,24^. The present study supported the cognitive reserve hypothesis^25^, which considers that a higher level of education increases neuronal plasticity and connectivity, as we document a significant protective effect of higher education level on AD, VaD, and MCI-VD prevalence. Although the characteristics of the study subjects showed that the level of social activities decreased as the prevalence of AD, VaD, and MCI-VD increased, logistic regression analysis showed that active social engagements are inversely related with an increasing prevalence of AD, VaD, and MCI-VD. This tendency suggested that regularly attending social activities may reduce the prevalence of CI among older subjects. Age, education, and social engagement have the same effects on the trend in prevalence of AD, VaD, and MCI-VD, whereas gender and single status (no spouse) differ in their influence on the prevalence of AD, VaD, and MCI-VD. Most studies^26–28^ show that the prevalence of AD is higher in women than in men, but the prevalence of VaD is lower in women than in men. Our data found that female gender is a risk factor associated with increasing prevalence of AD, but has the opposite effect for prevalence of VaD. Although it has been postulated that this may be related to estrogen, estrogen therapy did not improve cognitive function^29^. Our logistic regression analysis indicated that single status (no spouse) is a risk factor for increasing prevalence of AD, although it has no relationship with VaD. Our data indicated that female gender and single status (no spouse) were the risk factors associated with increasing prevalence of MCI-VD and AD. The best way to differentiate MCI-VD outcomes would be to follow-up the MCI group.

In the 2015 cohort, there was a lower rate of obesity but higher rates of vascular risk factors including heart disease, diabetes, and hypertension. Obesity, diabetes, and hypertension were risk factors for the increasing prevalence of VaD and MCI-VD. Heart disease was a risk factor for MCI-VD. In agreement with most previous research^30–32^, hypertension was a risk factor for the increasing prevalence of AD, VaDand MCI-VD. This may be because hypertension reduces cerebral metabolism, exacerbates arteriosclerosis and indirectly reduces acetylcholine^33,34^.

Some studies^35–37^ found that smoking was a risk factor for dementia. However, our logistic regression analysis did not detect a significant association of this factor with the prevalence of AD, VaD or MCI-VD. As is well-known, smoking is a risk factor of stroke, and increasing the cerebrovascular atherosclerosis which may be contribute to the risk of vascular dementia. However, some study^37^ found that former smoking and smoking less than 1 pack per day did not increase the risk of VaD. Our study did not distinguish former and current smoking, did not distinguish how much smoking may lead to smoking has no association of VaD and MCI-VD. It was suggested that the subjects in the 2015 cohort likely consumed more alcohol than those in the 2010 cohort. Logistic regression analysis found that alcohol consumption was a protective factor for the prevalence of AD, VaD and MCI-VD. Most of the subjects reported occasional or moderate alcohol consumption. A previous study showed that moderate alcohol consumption may reduce the prevalence of dementia, whereas heavy drinking is harmful for cognitive function^38^.

In summary, this study evaluated changes in cognitive impairment in a northern region of China from 2010 to 2015, and found that the prevalence of CI increased during these 5 years. To reduce the burden of dementia, the government should support more social investment to attenuate the increasing prevalence of cognitive impairment. The survey suggested that improving the educational level, increasing social activities, preventing and curing vascular risk factors, and reducing heavy alcohol consumption could reduce the prevalence of cognitive impairment. Our logistic regression analyses identified a significant difference between the 2010 and 2015 data when all factors were adjusted. This suggested that there were other factors influencing the increasing prevalence of cognitive impairment. Our study had certain limitations; we did not investigate genetic factors, other cognitive impairment risk factors such as anemia and hypercholesterolemia, hyperhomocystinemia, obstructive sleep apnea syndrome, and environmental factors due to necessary study limitations. Future work should evaluate these factors and compare their effects with the currently determined risk factors for cognitive impairment.
